# A Systematic Review of Apicomplexa Looking into Epigenetic Pathways and the Opportunity for Novel Therapies

**DOI:** 10.3390/pathogens12020299

**Published:** 2023-02-11

**Authors:** Yara de Oliveira Brandão, Marcelo Beltrão Molento

**Affiliations:** Graduate Program in Microbiology, Parasitology, Pathology, Laboratory of Veterinary Clinical Parasitology, Federal University of Paraná, Curitiba 80035-050, PR, Brazil

**Keywords:** parasite infection, *Toxoplasma gondii*, *Cryptosporidium parvum*, malaria, miRNA, non-coding RNA, DNA methylation, epidrugs, histone modification, protist

## Abstract

Interest in host epigenetic changes during apicomplexan infections increased in the last decade, mainly due to the emergence of new therapies directed to these alterations. This review aims to carry out a bibliometric analysis of the publications related to host epigenetic changes during apicomplexan infections and to summarize the main studied pathways in this context, pointing out those that represent putative drug targets. We used four databases for the article search. After screening, 116 studies were included. The bibliometric analysis revealed that the USA and China had the highest number of relevant publications. The evaluation of the selected studies revealed that *Toxoplasma gondii* was considered in most of the studies, non-coding RNA was the most frequently reported epigenetic event, and host defense was the most explored pathway. These findings were reinforced by an analysis of the co-occurrence of keywords. Even though we present putative targets for repurposing epidrugs and ncRNA-based drugs in apicomplexan infections, we understand that more detailed knowledge of the hosts’ epigenetic pathways is still needed before establishing a definitive drug target.

## 1. Introduction

Parasites and hosts have experienced an intense coevolution process [1]. Both genetic and epigenetic changes have occurred, leading to the rise of more resilient hosts and more virulent or adapted parasites [1]. The epigenetic concept came to light in 1942 with Waddington’s epigenetic landscape [2]. This concept proposed that the environment played an essential role in gene regulation, connecting the genotype to the phenotype, which could enlighten embryo development and cell differentiation. Later, the epigenetic concept underwent many interpretations and modifications, resulting in the current definition. Therefore, molecular epigenetics is an alteration in gene expression due to molecular mechanisms other than the change in the nucleotide sequence, such as histone chemical modifications, changes in chromatin structure, and non-coding RNA (ncRNA) [3].

The coordinated action of post-translational modifications of histones (PTHM), histone variants, DNA methylation, chromatin remodeling complexes, and ncRNAs (i.e., micro-RNA—miRNA, long non-coding RNA—lncRNA, and piwi RNA—piRNA) promotes changes in the chromatin structure that can allow or prevent gene transcription. In addition, ncRNAs can regulate gene expression at a post-transcriptional level by mRNA degradation or translation blockage [4]. These epigenetic mechanisms implicate the development and progression of many pathologies, such as cancer and neurological, metabolic, immunological, and infectious diseases in human and domestic animals [5,6]. Thus, enzymes that mediate the epigenetic gene regulation pathways have been used as drug targets, and epidrugs are currently used in clinical practice to treat hematological disorders [7].

Azacitidine and decitabine are examples of epidrugs that target epigenetic activity. These drugs inhibit DNA methyltransferases (DNMTs), which add methyl groups in CpG locations of DNA, leading to DNA methylation. Other enzymes responsible for epigenetic modifications are the writers and erasers that mediate histone marks by adding (e.g., histone acetyltransferase (HAT) or histone methyltransferase (HMT)) or removing (e.g., histone deacetylase (HADC) or histone demethylase (HDM)) chemical groups from histone tails. Histone deacetylases inhibitor (HDACi) drugs (e.g., vorinostat, romidepsin) and HMTi tazemetostat are approved by the Food and Drug Administration (FDA) to treat some types of lymphoma, while other HMT inhibitor (HMTi) drugs and HDM inhibitor (HDMi) drugs are in preclinical or clinical trial phases [8]. In addition to their therapeutical potential, epigenetic marks have been investigated for use in diagnosis. For example, DNA methylation of specific genes is already used for prostate and colon carcinoma diagnosis [7]. However, despite the advances in epigenetic-targeted therapy and diagnosis, no epigenetic-based drug or biomarker is available for parasitic diseases.

Parasitic diseases of humans and animals include the Apicomplexa phylum, such as *Toxoplasma gondii*, *Cryptosporidium* spp., *Plasmodium* spp., and *Theileria* spp. These unicellular, eukaryotic, obligate intracellular parasites reside in the parasitophorous vacuole (PV) derived from the host cell membrane (except for *Theileria* spp.). Their virulence factors are responsible for host cell adhesion, invasion, and the modulation of the host’s immune response and metabolism [9]. The success of parasites’ invasion, survival, and reproduction relies on their close interaction with the host’s genome, which has been demonstrated to implicate epigenetic mechanisms [10]. For example, parasites can induce chromatin remodeling in genes related to the host immune response, changing their expression and leading to a more beneficial environment for their development. In addition, parasites may modulate the expression of miRNAs involved in cell cycle regulation, aiming to keep the infected cell alive and allowing the parasite to survive and proliferate [10].

Given the importance and the therapeutic potential of the epigenetic pathways employed by Apicomplexa in infected host cells, this review aims to carry out a bibliometric analysis of the publications related to host epigenetic changes during apicomplexan infections and to summarize the main studied pathways, pointing out those that represent putative drug targets. Understanding which host pathways apicomplexan parasites modulate and how they do it can unravel new drug targets.

## 2. Material and Methods

### 2.1. Information Sources and Search Strategy

The protocol of this study was registered in the International Prospective Register of Systematic Reviews (PROSPERO: http://www.crd.york.ac.uk/prospero, accessed on 25 January 2023), CRD42023391155.

We screened relevant reports in Scopus, Science Direct, Web of Science, and PUBMED databases. In Scopus, PUBMED, and Science Direct, we searched under “title, keywords, and abstract”; in Web of Science, we searched under “topic”. All searches were limited to “article” (Web of Science and Scopus), with the following search term combinations: epigen* AND (“parasitic diseases” OR parasite OR protozoan); (“DNA methylation” OR “histone modification” OR “non-coding RNA”) AND (“parasitic diseases” OR parasite OR protozoan); epigen* AND parasite AND host; epigen* AND (“host gene” OR “host response” OR “host cell”) AND (“parasitic diseases” OR parasite OR protozoan); “chromatin remodeling” AND (“parasitic diseases” OR parasite OR protozoan); (“micro RNA” OR “miRNA” OR “lnc RNA” OR “small non-coding RNA” OR “long non-coding RNA” OR “piwi RNA” OR “pi RNA”) AND (“parasitic disease” OR parasite OR protozoan); (“modification* of histone*” OR “histone* modification*”) AND (“parasitic diseases” OR parasite OR protozoan); “DNA methylation” AND (“parasitic diseases” OR parasite OR protozoan). As the Science Direct database does not accept wildcards, the words “epigenetic”, “modification”, and “histone” were applied instead of “epigen*”, “modification*”, and “histone*”. The searches were performed in August 2020 and the last update was made in October 2022. Papers listed as a reference in selected articles were also evaluated. We carried out two new searches in January 2023, using the same keywords and replacing the term “protozoan” with “protist*.” However, no paper found exclusively under the “protist” term fulfilled the inclusion criteria, and we retained the results of the search that was carried out in October 2022.

### 2.2. Search Analysis—Eligibility Criteria

1. Research article: only original research articles were included.
2. Epigenetic approaches: papers showed one or more tests to demonstrate the epigenetic event.
3. Host–parasite interaction: studies investigating modifications due to the interaction between the parasite and the host.
4. Host species: only studies in mammalian hosts were included.
5. Parasite species: only Apicomplexa parasites were included.
6. Time period: articles were included independently of the year of publication.
7. Idiom: articles published in any language were considered. For those published in idioms other than Portuguese or English, Google Translate (https://translate.google.com/, accessed on 25 January 2023) was used for translation into English.

### 2.3. Study Records, Data Items, Outcome, and Bias Risk Evaluation

Title, abstract, and keywords information were obtained in RIS format and imported to the reference manager Mendeley Desktop 1.19.8 software (Mendeley Ltd, London, UK). After excluding duplicated documents, the articles were screened by analyzing the title and abstract, addressing the inclusion criteria. The selected papers were fully accessed, and those not fulfilling the inclusion criteria were excluded. A Microsoft Excel table was filled with information regarding parasite species, the gene expression regulatory mechanism, the targeted process, the research models, the country, and the complete reference. In the topic “regulatory mechanism,” the type of event was recorded: i.e., DNA methylation, post-translational modifications of histones, or ncRNA (miRNA, lncRNA, piRNA, circRNA). The “targeted process” refers to the main cell function pathways cited in each study. To assess the bias risk, title, abstract screening, and full paper accessing were carried out by the two authors independently.

### 2.4. Additional Analysis

For keyword and country co-occurrence analysis, the selected papers were imported to VOS Viewer 1.1.16 (Leiden University, Leiden, The Netherlands). A world map with the distribution of the selected reports was constructed using Microsoft Excel software.

## 3. Results and Discussion

### 3.1. Document Selection

**Figure 1:**
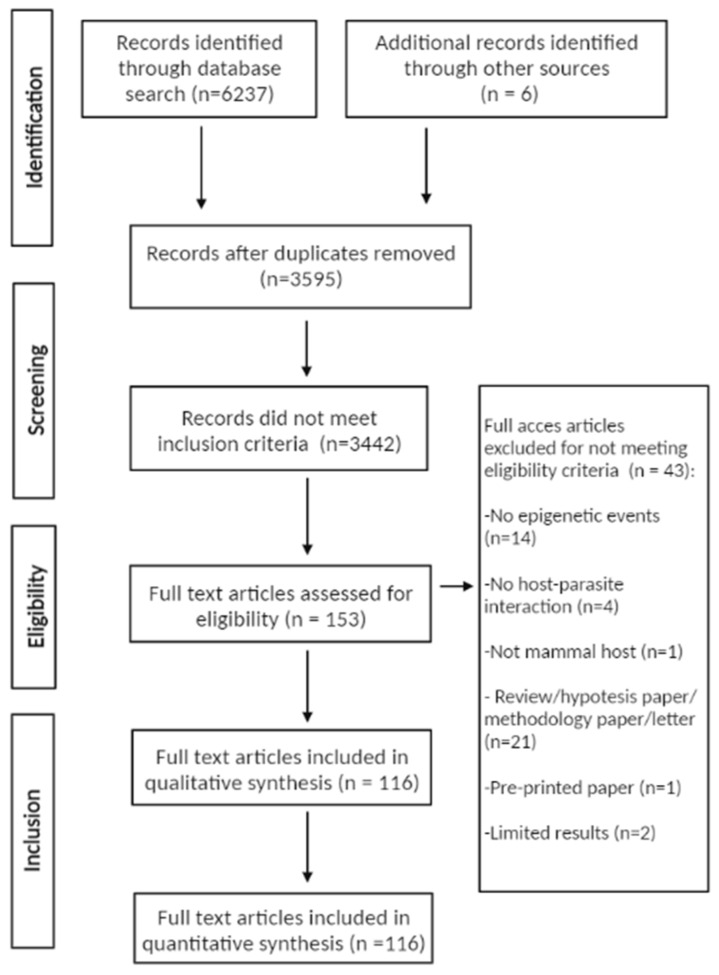
Flow chart of returned results from the present database search.

The search returned 6237 documents, of which 3595 remained after duplicate exclusion. Upon screening the title and abstract, 3442 records did not meet the eligibility criteria. Of the 153 remaining records that were fully accessed, 43 did not meet the eligibility criteria. Six docs were added from the references of fully accessed articles, resulting in a total of 116 included records (Figure 1). Of the 43 excluded studies, 14 did not present epigenetic events; four reported epigenetic events that were not related to the host-parasite interaction; one study investigated a host that was not a mammal; two studies did not show sufficient evidence; one study was a pre-printed article; 21 studies were not research articles (i.e., opinion letters, methodology or hypothesis papers, case reports or addenda to articles, and 16 review articles).

### 3.2. Analysis of Affiliated Countries

**Figure 2:**
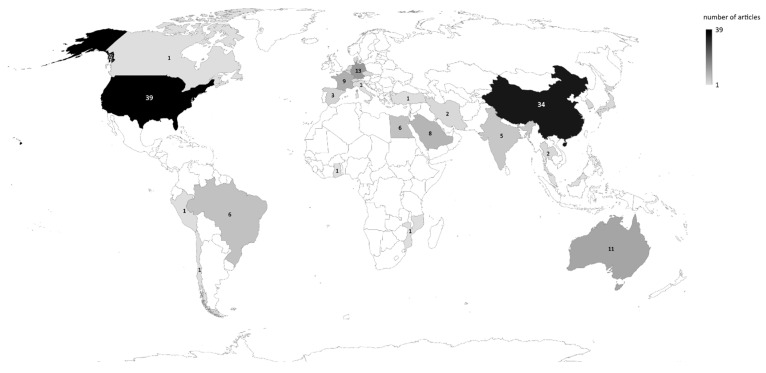
Map of the distribution of publications by country. The color intensity varies according to the number of publications.

**Figure 3:**
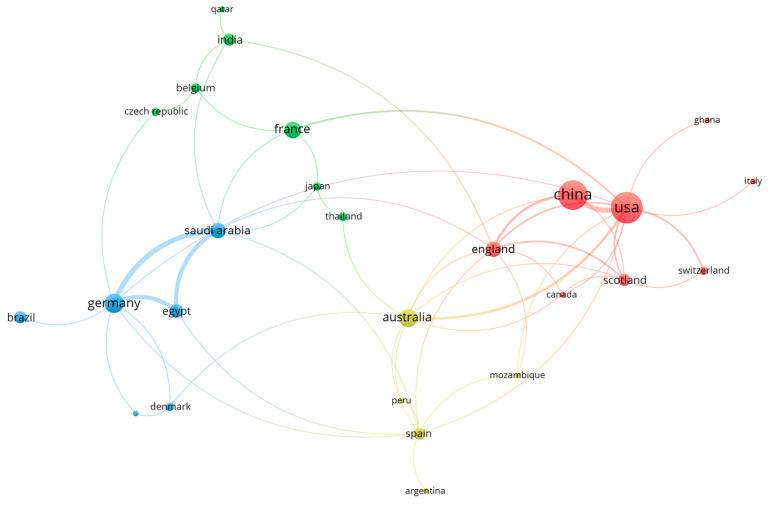
Co-occurrence network by country. The cluster (different colors) is formed by the frequency of the country’s names co-occurring, as the node size is proportional to the number of pub-lished articles. The blue cluster displays the countries most cooperated with Germany (Saudi Arabia, Egypt, Denmark, Brazil, and the Netherlands – node not labeled). The green cluster is composed of countries that most collaborated with France, and in the yellow cluster, the collaborations with Australia. In the red cluster, it is displayed the countries that cooperated with USA and China. The line thickness is proportional to the number of articles published in collaboration between countries.

Table 1 includes all collected articles showing a limited range of parasites, possible regulatory mechanisms, target processes, and research models. The United States of America (USA) had the highest number of publications on the topic of this review (33.6%), followed by China (29.3%) (Figure 2). Those two countries also showed strong cooperation in paper publications (Figure 3). In the country co-authorship analysis, the closer the node, the higher the cooperation. Countries with the same node color were included in the same cluster, which was formed by the frequency of the co-occurrences of the countries’ names. The node size depicts the number of publications (the larger the node, the higher the number of publications), and the thickness of the lines represents the number of collaborations. China and the USA had the largest nodes, belonging to the same cluster and linked by a thick line. These characteristics reflected the findings above: i.e., many publications and strong cooperation.

Interestingly, the most representative countries in this review (USA, China, and Germany) did not present the highest prevalence of Apicomplexan-related diseases. For example, a recent meta-analysis showed IgG seroprevalence against T. gondii in less than 10% of pregnant women in the USA, compared to more than 50% in low and middle-income countries (LMICs), such as Brazil [127].

The same pattern was also observed to concerning malaria and cryptosporidiosis [13,14,128,129]. The lack of studies in the most-affected countries was related to their socioeconomic status and research and development investments [15]. Diseases such as cryptosporidiosis and toxoplasmosis can be prevented by having access to clean water and education, which is not a reality for most of the populations in LMICs [14]. Therefore, these countries cannot guarantee ways to prevent these diseases or support quality research. The lower research investment in LMICs reflects the smaller number of publications. However, this could be enriched by more research collaborations between the North and South.

### 3.3. Keywords Co-Occurrence Analysis

**Figure 4:**
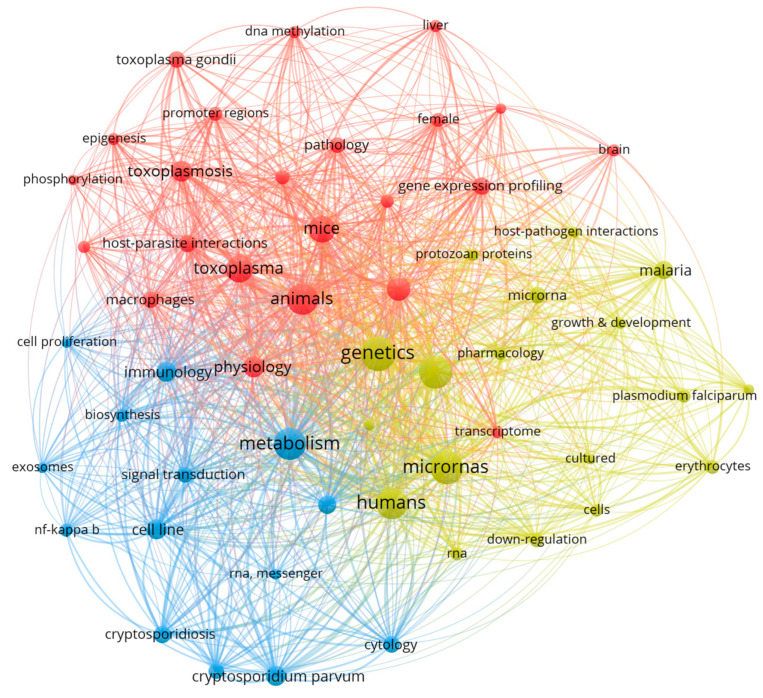
Co-occurrence network by keyword segregated according to their occurrence in colored clusters (red: words related to *T. gondii*; blue: *C. parvum*; yellow: *Plasmodium* spp.). The node size is proportional to the word occurrence among the studies. Linked nodes represent the co-occurrence of the keyword in articles.

The keywords co-occurrence network analysis resulted in three clusters (Figure 4). The clusters are identified by different colors showing words related to the findings presented in Table 1 and summarized in Table 2. The red cluster displays the words related to *T. gondii*, the most represented parasite. *T. gondii* is an intestinal coccidian with felids as definitive hosts, infecting a wide variety of avian and mammal (i.e., mice and humans) intermediate hosts. In its acute phase, *T. gondii* invades and replicates into the host cells, especially macrophages, causing mild clinical signs. In the chronic phase, tissue cysts are responsible for neurological and ocular disorders in offspring through congenital transmission [130]. The significant number of studies concerning *T. gondii* may be due to its pathogenicity, high prevalence in humans, and worldwide distribution. Other reasons are that tachyzoites are successfully maintained in culture, are tractable for genetic manipulation, and evoke a vigorous immune response in the host; therefore, they are used as models for apicomplexan parasites studies [127,131]. However, despite the number of articles selected, only three studies used samples from toxoplasmosis patients [91,106,107], and only one article investigated *T. gondii* in its definitive host [90]. Although felids usually do not present severe clinical signs associated with toxoplasmosis, they are an important contamination source; thus, understanding how the parasite manipulates the felid epigenetics would be of great value.

The second and third most cited parasites were *Plasmodium* spp. and *Cryptosporidium parvum.* They are represented in the yellow and blue clusters, respectively. Malaria, a disease caused by *Plasmodium* spp., was estimated to account for 229 million cases and 409.000 deaths in 2019, according to the World Malaria Report of the World Health Organization [128]. Despite its epidemiological importance, the number of studies of *Plasmodium* spp. was a bit more than half of the number of studies of *T. gondii*. This low number of reports may have three possible reasons: (1) the characteristic of the inclusion criteria (as we excluded studies in invertebrate hosts, such as *Plasmodium* spp. Vectors); (2) difficulties in maintaining this parasite in culture, which often restricted clinical sampling in experiments [132]; and (3) the infected cell type of the host cell. The mature erythrocyte is one of the targets of *Plasmodium* spp. but it lacks a nucleus, DNA, and chromatin. Thus, DNA methylation, post-translational histone modifications, chromatin remodeling, and change in miRNA expression cannot occur in these cells, regardless of the infection. The selected *Plasmodium* spp. studies focused on the epigenetic modifications in leukocytes, other infected cell types (i.e., hepatocytes), ncRNA in the plasma, and the ncRNA content of extracellular vesicles secreted by infected erythrocytes. Therefore, more studies are needed covering the possible effects of *Plasmodium* spp. infection in epigenetic events during the erythropoiesis process. These modifications can, for example, be responsible for alterations in the mature erythrocytes, such as the microRNA composition of the secreted exosomes [58].

*C. parvum* is an intestinal protist responsible for epithelial barrier disruption and enterocyte apoptosis. These lesions result in nutrient malabsorption and persistent diarrhea [133]. The disease is more severe in immunocompromised human patients and young animals; however, none of the selected studies investigated the *C. parvum*-related epigenetic changes in these individuals. Eight studies use in vivo models, and none included samples from human patients or domestic animals. One probable limitation for studies with clinical samples is the strict location of the parasite in the intestine, making sample collection difficult. However, intestinal tissue from biopsies or even the extraction of genetic material from feces could be used to investigate the epigenetic modifications during acute cryptosporidiosis. Other words highlighted in the co-occurrence analysis, such as “microrna”, “dna methylation”, “phosphorylation”, “transcriptome”, and “gene expression profiling”, were related to the epigenetic regulation of gene expression. ncRNAs were the targets of most of the studies, followed by PTHM and DNA methylation (Table 2).

In recent decades, the interest in ncRNAs, such as miRNA, lncRNA, siRNA, and piRNA, has increased considerably. The next-generation sequencing (NGS) technology, and therefore RNA sequencing (RNAseq), has enabled the investigation of the total transcriptome and the discovery of many ncRNAs [134]. Fifty-one of the 88 ncRNAs studies (58%) focused on identifying the different profiles of ncRNA expression in multiple cell lineages and host species infected by apicomplexan parasites, covering a high number of transcripts. The high specificity of miRNAs and lncRNAs for cell type and host species highlights the importance of these studies [134]. However, it is crucial to clarify the role of ncRNA in each parasite species before suggesting new targets for drugs or biomarkers for diagnosis. The lower number of studies of ncRNA function (42%) confirmed the relatively recent interest in this RNA in apicomplexan infection [46]. We expect that the ncRNAs identified by the studies on the difference of expression will be further investigated to unravel their pathways in apicomplexan infections. The co-occurrence network of keywords also showed that the different apicomplexan parasites (*T. gondii*, *Plasmodium* spp., and *C. parvum*) drive epigenetic changes in the same host pathways. Words such as “immunology”, “cell proliferation”, and “nf kappa-B” displayed in the blue cluster were linked to nodes in red and yellow clusters, referring to the host process that suffered interference from the parasite (Table 1). As expected, the host defense was the most epigenetically influenced route. In healthy individuals, infections elicit an immune response by activating pathways regulated by epigenetic mechanisms. In addition to activating these defense pathways, the apicomplexan parasites may drive changes in epigenetic events to evade the immune response and guarantee their survival. The parasite preserves its habitat by interfering in apoptosis regulation, favoring its survival and reproduction.

Epigenetic changes are also related to the pathogenesis of apicomplexan parasites infection. For example, the pathogenic B cell proliferation in *Theileria annulata* infection can be related to changes in miRNA expressions that regulate cancer pathways. Epigenetic changes in cell migration in *C. parvum* infection are related to the lesion and clinical signs associated with the disease [23,69]. Multiple pathways share many of the epigenetic mechanisms reported here. Changes in miRNAs expression and the activation or inhibition of enzymes related to DNA methylation or PTHM can alter multiple routes. Thus, future studies are needed to identify the exact pathways modulated by parasites to ensure the specificity of putative drug targets.

### 3.4. Epidrugs: Drug Targets and Host-Directed Therapy

The current treatment for apicomplexan parasite infections relies on drugs directed at parasite-specific molecules or pathways essential for their survival or replication. The rapid reproductive life cycle of the organism favors the emergence of mutations, leading to drug resistance that is not a major problem in host-directed therapy (HDT) [135]. Another benefit of HDT is the possibility of repurposing preapproved drugs. HDT may also activate or modulate host pathways, such as innate and adaptative immune responses, inflammation, cell cycle regulation, and apoptosis [136]. Although epidrugs aimed at the parasite have been suggested [137], studies on the use of these drugs to modulate host response are still lacking (Table 3; Supplementary Table S2). Understanding which host pathways are epigenetically modulated by apicomplexan parasites and how they do it can unravel new drug targets. In this section, we summarize the current knowledge regarding epigenetic modifications induced by Apicomplexan parasites and highlight putative targets for repurposing epidrugs.

#### 3.4.1. Histone Post-Translational Modifications and Chromatin Remodeling

Drugs targeting PTHM enzymes HDAC, HMT, and HDM were previously approved for cancer treatment or were in pre-clinical or clinical trials. Here, we describe the host process that could be related to these drugs in apicomplexan infections.

**Figure 5:**
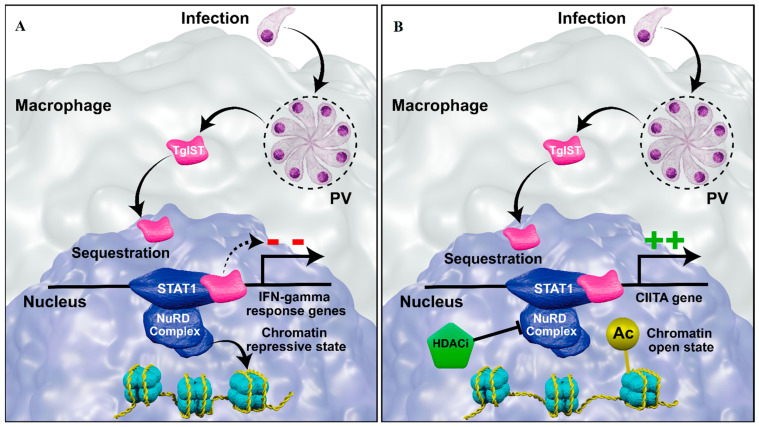
Modulation of IFNGγ response genes in macrophages upon *T. gondii* infection (**A**) and in infected macrophage treated with HDACi (**B**). (**A**) *T. gondii* tachyzoite replicates in the parasitophorous vacuole (PV), releasing the *T. gondii* inhibitor of STAT1 transcriptional activity (TgIST) (pink), which is translocated to the nucleus. TgIST sequesters STAT1 and recruits the Mi2/NurD complex to the promoter of IFNγ response genes, remodeling the chromatin to a repressive state, thus silencing the gene. (**B**) The HDACi inhibits the action of the histone deacetylase (HDAC) present in the Mi2/NurD complex. The inhibition of HDAC prevents the histone deacetylation and leads to an open chromatin state, restoring the *Ciita* gene expression (an IFNγ response gene).

*T. gondii* infection epigenetically modulates the IFNγ, NFκB, and TNFα pathways, which interfere with monocyte differentiation and activation [105]. For example, in murine macrophages, *T. gondii* secretes the effector *T. gondii* inhibitor of STAT1 transcriptional activity (TgIST), which reaches the nucleus and complexes with STAT-1 (signal transducer and activator of transcription 1) and Mi-2/NuRD, repressing the transcription of IFNγ response genes [96] (Figure 5A). The exact mechanism of the TgIST-induced gene-expression inhibition has not been completely elucidated; however, the gene-expression regulation seems not to be the same for all IFNγ responsive genes. Lang et al. (2012) [75] observed a decrease in the histone H3 acetylation associated with a lower expression of the secondary IFNγ response gene Ciita, which was restored after treatment with MS-275. Entinostat, or MS-275, which is an HDAC1 inhibitor with a lesser action in HDAC2 and 3 and was in a clinical trial for breast cancer treatment. The intracellular pathogen *Mycobacterium tuberculosis* also silenced the Ciita gene through H3 deacetylation, and treatment with sodium butyrate partially rescued the gene expression [138]. In addition to MS-275, the FDA-approved drugs vorinostat and romidepsin inhibit HDAC1 and 2 and could be tested to restore Ciita and other secondary IFNγ response gene expression, increasing host immune defense (Figure 5B). Moreover, these HDACi enzymes may rescue TNFα expression, whose gene silencing function seems to be due to a decrease in H3 acetylation in *T. gondii*-infected macrophages [72].

The toxoplasma E2F4-associated EZH2-inducing gene regulator (TEEGR) is another *T. gondii* granule that reaches the host cell nuclei, inducing epigenetic modifications. In HFF and BMDM cells, this effector was demonstrated to recruit the histone methyltransferase enhancer of zeste 2 polycomb repressive complex 2 subunit (EZH2) to the promoter of NFκB-dependent genes, increasing the histone closing mark H3K27me3, thereby repressing the expression of interleukin 8, 6, and 1β. The negative modulation of these genes contributes to parasite persistence in the host [97]. The parasite *Leishmania donovani* uses a similar mechanism in mice macrophages to silence the inflammation-related gene *inos.* The in vitro and in vivo knockdown of EZH2 followed by *L. donovani* infection restored the iNOS expression [139]. The FDA-approved drug tazemetostat is a selective HMTi that specifically inhibits EZH2 and is also a promising epidrug option to restore the expression of NFκB pathway genes that are inhibited in *T. gondii* infection.

Post-translational modifications in histones have been determined in HCT8 cells and the intestinal cells of mice infected with *C. parvum* [28,35]. *C. parvum* transcripts were demonstrated to interact with the methyltransferase G9a, promoting the trimethylation of H3K9 and the consequent silencing of the immune response, cell differentiation, cell adhesion, and migration-related genes [22,23,24]. Therefore, the hypothesis that G9a methyltransferase inhibition in *C. parvum* infection decreases cell damage, boosts cell differentiation, and increases enteric epithelium repair should be investigated. When confirmed, the G9a may be a target for the HMTi drug for cryptosporidiosis treatment.

Mathy et al. (2022) [34] showed an increase in the expression of the lncRNA Nostrill in IEC4.1 cells and the intestinal cells of mice infected with *C. parvum*. This lncRNA mediates the increment of the open H3K4me3 mark and the recruitment of RNA polymerase II to the promotor of the gene *Ifr7*, increasing this gene expression, which is a regulator of the I-IFN pathway. Further inquiry on the role of this lncRNA in *C. parvum* may reveal novel targets for ncRNA-based therapy.

The active histone mark H3K4me3 has been monitored in peripheral blood monocytes from malaria patients. Genes related to innate immune memory response (trained immunity), such as interleukin 6 (IL6) and genes of the PI3K/AKT/mTOR pathway, showed increased levels of H3K4me3 and higher expression in monocytes primed with *P. falciparum* and stimulated with LPS when compared to control. This condition suggests that specific HMTs are involved in the development of immune memory in malaria [54,56]. Moreover, no differences were found in histone marks H3K27ac (open), H3K4me (open), and H3K9me3 (repressive) in innate memory genes of bone marrow monocytes from mice infected and reinfected with *P. chabaudi* [45]. These findings suggest that the trained immunity in *Plasmodium* spp. may be associated with epigenetic events, and *Plasmodium* species and cell lineage should be considered. Elucidating the innate immune response memory mechanism in malaria and the pathways involved in host tolerance to *Plasmodium* spp. will help in identifying targets for immunomodulators that can increase the host response to the parasite and avoid severe inflammation and tissue damage.

**Figure 6:**
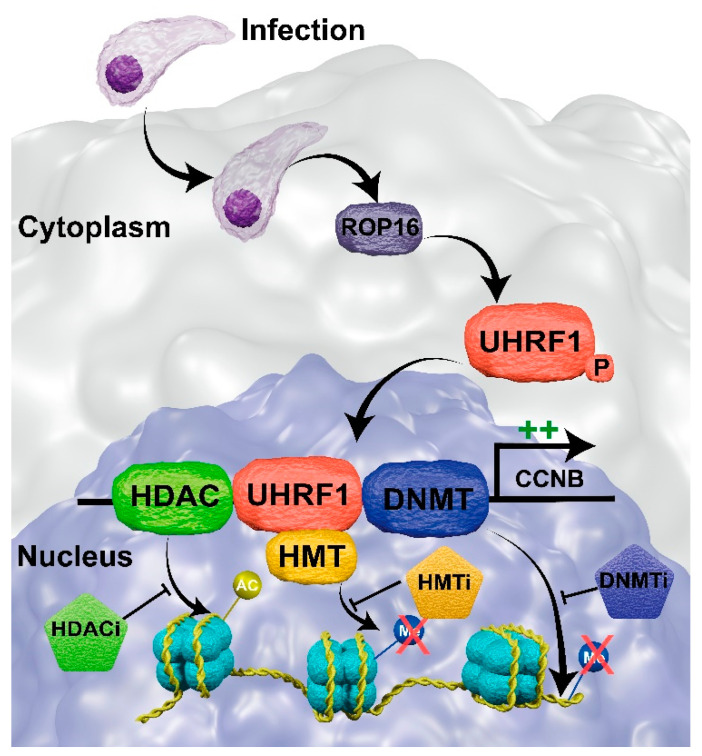
Epigenetic regulation of *CCNB* gene in BeWo cells upon *Toxoplasma gondii* infection and the mechanism of inhibition by putative drug targets. *T. gondii* secretes the effector Ropthry 16 (ROP16) in the cytoplasm of the infected cell that phosphorylates the ubiquitin-like containing PHD and RING finger domain (UHRF1) that translocates to the nucleus. The UHRF1 recruits the histone deacetylase (HDAC), histone methyltransferase (HMT), and DNA methyltransferase (DNMT) enzymes to the *CCNB* promoter, silencing its transcription and arresting the cell cycle in the G2 phase. We hypothesize that treatment with histone deacetylase inhibitor (HDACi), histone methyltransferase inhibitor (HMTi), or DNA methyltransferase inhibitor (DNMTi) can prevent histone H3 deacetylation, H3K9 methylation, and DNA methylation, respectively. It would restore the cyclin B expression and the continuity of the cell cycle.

PTHM and chromatin remodeling were found to change gene expression related to processes other than immune response. In *T. gondii* infections of BeWo cells, the protein Ropthry 16 (ROP16) phosphorylates the ubiquitin-like containing PHD and RING finger domain (UHRF1), which is translocated to the nucleus. The UHFR1 recruits HDACs and HMTs, decreasing H3 acetylation and increasing the H3K9me3 mark in the cyclin B1 gene (*CCNB1*). In addition, UHFR1 recruits DNMT, increasing the CpG methylation in the *CCNB1* promoter [111]. Those epigenetic events result in gene silencing, leading to cell-cycle arrest in the G2 phase (Figure 6). It has been shown that the *T. gondii* proliferation rate is higher when the host cell is in the G2 phase rather than in the G1 phase [140], although the reason for that remains unknown. Further investigation into the importance of cell-cycle arrest for the *T. gondii* infection success may identify potential drug targets for HDACi, HMTi, or DNMTi.

#### 3.4.2. DNA Methylation

The methylation status of CpG sites is also responsible for chromatin remodeling and gene expression regulation. Usually, the hypomethylation of CpG in gene promoters is related to increased gene expression. This pattern was observed in the gene *Tlr6* in the liver of mice infected with *P. chabaudi*. The TLR6 (toll-like receptor 6) acts with TLR2 in the recognition of pathogen-associated molecular patterns (PAMPs) by macrophages. This finding suggests the role of DNA methylation in the recognition of pathogens by Kuppfer cells in the liver [43]. The tissue damage caused by inflammation in malaria and the relation between TLR6 and malaria severity in *P. falciparum* and *P. vivax* infections [141] enforces the need for further investigation of *Tlr6* and other genes of the *Tlr* family.

Changes in DNA methylation were also observed in the liver tissue of mice vaccinated against *P. chabaudi*. Some modifications observed in immune-related genes were the hypomethylations of *Cx3cl1* and *Gp130* genes, which are responsible for monocyte recruitment and IL-6 signal transducer, respectively. In addition, the hypermethylation of the *Socs1*, an inhibitor of the JAK/STAT pathway, was observed [44]. *Leishmania donovani* and *Helicobacter pylori* are two other intracellular pathogens that induce changes in the DNA methylation status of genes of the JAK/STAT pathway, downregulating or upregulating the immune response, respectively [142,143]. Taken together, these findings regarding DNA methylation in *Plasmodium* spp. infection suggest that DNMTi can be useful in the modulation of the host immune response and inflammation, although a deeper knowledge of the role of DNA methylation in malaria infection is needed.

The unwanted hypomethylation is important to consider in the use of DNMTi. Gupta et al. (2017) [51] demonstrated that the drug-resistance gene *ABCB1* is down-methylated in severe malaria patients’ leukocytes, resulting in higher expression of the ABCB1 protein in such patients compared to that in uncomplicated malaria cases and healthy individuals. Thereby, the use of DNMTi can increase resistance to anti-malarial drugs. Therefore, future studies aiming to find targets for DNMTi drugs for this global disease should be carefully planned, considering the possible side effects of the drug and the possibility of undesired hypomethylation of the *ABCB1* gene.

Epigenetic modifications driven by parasites also interfere in host reproduction and behavior. *T. gondii*-infected mice presented lower levels of luteinizing hormone (LH), testicular function, and sperm production than those of uninfected animals. DNA methylation analysis of testicular tissue revealed global DNA hypermethylation and hypomethylation of specific CpGs in the *Crem* and *Hspa1* genes and the *Creb1* promoter. As these genes are necessary for healthy spermatogenesis, their methylation and silencing could affect the reproductive fitness of infected animals [83].

Although T. gondii seems to interfere with testis function, testosterone levels are higher in infected mice. This hormone promotes hypomethylation and increases the expression of the arginine vasopressin (AVP) promoter gene in the poster dorsal medial amygdala (MePD), a region related to sexual behavior [84]. This effect leads to reduced fear and behavioral change in *T. gondii*-infected mice. Specifically, these mice become attracted to the odor of felines’ urine, favoring the perpetuation of the parasite’s life cycle [84,113].

The treatment with L-methionine, a methyl donor for DNA methylation, restored the fear of felines’ urine odor and reaffirmed the role of DNA methylation in behavior changes. L-methionine and S-adenosylmethionine, the major methyl donors, are marketed as dietary supplements in the USA, and they have been studied to treat metabolic and neurological disorders [144,145]. The medial amygdala is also part of the dopamine system. Syn et al. (2018) [92] reported that human retinal cells infected with *T. gondii* presented a reduced methylation and a higher expression of genes related to dopamine and amyloid pathways, which have an essential role in neurodevelopment and neurodegeneration.

Interestingly, these changes in behavior and in the neuronal pathways were not attributed to a localized effect of the *T. gondii* cysts, as was early suggested [146]. Whether the AVP, dopamine, and amyloid system changes are a consequence of a long-distance signaling process (i.e., testosterone-induced AVP hypomethylation and expression) or a local disturbance induced by *T. gondii* cysts remains unclear. Further study of the role of DNA methylation in *T. gondii* infection could help in identifying targets for epidrugs, with the aim of minimizing the undesirable neurological consequences of congenital toxoplasmosis.

#### 3.4.3. ncRNA

Several miRNAs, their targets, and their regulatory pathways have already been described. It is also known that some drugs targeting miRNAs are undergoing clinical trials [147]. Some of these targeted miRNAs, such as miR155 and miR21, are involved in apicomplexan infection. The miR-155 miRNA has been investigated in many studies of *Toxoplasma gondii* infection [79,93,99,104,106,107,114,115,119,121]. This miRNA was upregulated in the brain of mice infected with the type-II strain of *T. gondii* and was related to its chronic phase during the development of cysts [79]. A recent study demonstrated that miR-155 was present in exosomes secreted by dendritic cells in *T. gondii* infection [121]. These exosomes are ingested by murine macrophages, and the miR-155 targets the suppressor of cytokine signaling 1 (SOCS1), which is an inhibitor of the NFκB pathway, increasing the activation of this pathway and resulting in the control of *T. gondii* tachyzoites proliferation.

Although miR-155 can boost the immune response and help control *T. gondii* infection, the activation of immune and inflammatory pathways may also be harmful. Meira-Strejevitch et al. (2020) [106] found miR-155 to be upregulated in the plasma of ocular toxoplasmosis patients, when compared to the disease’s asymptomatic form. In this scenario, the increase in immune and inflammatory pathways driven by this miRNA seemed to contribute to the retinal lesion. This suggests that this miRNA is a potential target for ncRNA-based therapy that modulates the host immune response. Nonetheless, the acute or chronic disease phase may be considered to investigate whether a miR-155 mimic or an anti-miR-155 will achieve better clinical results. In the latter case, the repurpose of MRG-106, an anti-miR-155, is in a clinical trial for lymphoma treatment [147].

The gain of miR155 expression was found in murine Kupffer cells during *P. berghei* genetically attenuated parasite (GAP) infection. The induced overexpression of this miRNA in GAP pre-sensitized mice raised their protection against *P. berghei,* suggesting that an miR-155 mimic can boost the immune response against this parasite after the GAP vaccine [39]. Cell proliferation and cancer development are other pathways related to miR-155. The miR-155 miRNA was observed to be upregulated in bovine B cells infected with *T. annulata* and in its exosomes [69,70]. During *T. annulata* infection, miR-155 targets the light-mediated developmental protein (DET1), which is involved in the ubiquitination of the c-Jun protein that works as an oncogenic transcription factor. In addition to inducing cell proliferation, c-Jun promotes positive feedback for miR-155 expression. The sustained miR-155 expression results in the maintenance of cell proliferation and the *T. annulata* cancer-related development. Considering the important role of miRNA in *T. annulata* infection, drug testing (i.e., MRG-106) should be carried out for developing new cattle treatment.

The miR-21 miRNA is also targeted by drugs that are undergoing clinical trial [147], and it seems to play a key role in apicomplexan infection. Like miR-155, miR-21 is known for its role in cancer development, characterized as an oncomiR. In colorectal cancer cells infected with *C. parvum*, miR-21 was found to be upregulated, suggesting that this parasite can play a critical role in oncomiR regulation, and thus in cancer development and evolution [25].

The miR-21 miRNA is also implicated in immune-response modulation as an anti-inflammatory molecule. The lower expression of this miRNA in the plasma of ocular toxoplasmosis patients, compared to that of asymptomatic individuals, may contribute to ophthalmic lesions [106]. On the contrary, this miRNA was found to be upregulated in the intestine of mice infected with *C. parvum*, where the miR-21 targeted the chemokine CCL20, diminishing the host defense against the parasite [21]. Moreover, these findings demonstrate that miR-21 plays a regulatory role in the immune response in apicomplexan infection, and its overexpression or silencing will vary according to the condition. The miR-21 miRNA was upregulated in the *E. papillata* infection of mice, but its role during the disease was not further investigated [126]. Although miR-21 may influence cell proliferation and immune response in apicomplexan infection, its target and the pathways need to be better described.

Other miRNAs have been demonstrated to have important roles in apicomplexan infection and should also be considered future drug targets. One of these miRNAs is miR-451, which is present in extracellular vesicles (EVs) secreted by red blood cells. EV secretion is increased in *P. falciparum* infection, and the EVs are internalized by endothelial cells and by the parasite [52]. In the endothelial cells BMEC-1, miR-451 targets CAV-1 (caveolin-1) and activates the transcription factor 2 (ATF2), downregulating their expression and resulting in endothelial barrier dysfunction [48]. In *P. falciparum*, miR-451 recognizes the 3′ and 5′ UTR region of *var* genes transcripts, blocking their translation and downregulating the expression of *P. falciparum* erythrocyte membrane protein-1 (PfEMP1), which can play a crucial role in host cell adhesion and invasion [52]. In addition, miR-451 targets the transcript of the regulatory subunit of the cAMP-dependent protein kinase (PKA-R) of *P. falciparum*, reducing its translation and suppressing parasite development. This mechanism seems to confer resistance against plasmodial infection in sickle cell disease patients [47]. Therefore, miR-451 is a great potential target for drugs that aim to control the parasite burden and endothelial cell damage.

The role of miRNAs of the let7 family has also been studied in apicomplexan diseases in *C. parvum* infection. Studies of cholangiocytes (H69 cells) and enterocytes (IEC4.1 cells) showed that during *C. parvum* infection, the toll-like receptor 4 (TLR4) is activated, leading to the stimulation of the NFκB pathway [16,22]. In H69 cells, the NFκB p50 is responsible for forming a silencer complex that deacetylases the histone H3 in the let7i promoter, inhibiting the expression of this miRNA [14]. The let7i targets several components of the immune response, such as TLR4, the cytokine-inducible Src homology 2-containing protein (CIS), and sirtuin-1 (SIRT1) [11,15,20]. Thus, the inhibition of let7i expression increases these proteins, which act as regulators of the immune response pathway. The increase in TLR4 results in an increment of NFκB, which inhibits let7i expression, showing positive feedback for TLR4 and NFκB expression [11]. The CIS protein stimulates the NFκB through the degradation of IκBα (NF-kappa-B inhibitor alpha) and inhibits inflammatory cytokines [15]. The inhibition of let7i also stimulates NFκB expression through CIS activity. However, the downregulation of let7i increases SIRT1, an inhibitor of NFκB [20]. These findings suggest that let7 family miRNAs promote a fine-tuned regulation of immune response, mainly through the NFκB pathway in cryptosporidiosis. Thus, manipulating this miRNA could increase the immune response against *C. parvum*.

## 4. Summary

Our study presented a bibliometric analysis of selected reports on apicomplexan parasite-driven epigenetic changes in hosts. It also summarized the main findings in the literature, pointing out the pathways that could be addressed by drugs targeting histone PTM, DNA methylation, and ncRNAs. The repurposing of drugs in the HDT context may be a shortcut for the implementation of new treatments for parasitic diseases. Herein, we presented putative targets for novel epidrugs and ncRNA-based therapies in apicomplexan infections. However, we understand that more detailed knowledge of the hosts’ epigenetic pathways addressed by the apicomplexan parasites is still needed before establishing a definitive drug target. Although the number of studies in this context is rather limited, the rising number of publications in the past decade reveals an increasing interest in the scientific community about the role of epigenetics in apicomplexan diseases. Moreover, we believe that the emergence of novel therapy options could be enhanced through drug repurposing. The diseases presented here cause major social and health problems globally, and we have the opportunity to improve human and animal health and welfare by consolidating international collaborative work.

## Figures and Tables

**Table 1:** List of the articles included in this review related to the parasite, regulatory mechanism, target process, research model, and country of publication.

Parasite	Regulatory Mechanism	Target Process	Research Model		Country	Ref.
			In Vitro/In Silico	In Vivo		
*C. parvum*	miRNA	Host defense	H69 cells	-	USA	[11,12,13,14]
*C. parvum*	miRNA	Host defense	H69 and HIBEpiC cells	-	USA	[15,16]
*C. parvum*	miRNA	Cell proliferation	H69 and HIBEpiC cell lines	-	USA, China	[17]
*C. parvum*	miRNA	Host defense	H69, HIBEpiC, 603B, IEC4.1, SW480 cells	C57BL/6J mice	USA	[18]
*C. parvum*	miRNA	Host defense	H69, 603B, IEC4.1	C57BL/6J mice	USA	[19]
*C. parvum*	miRNA	Host defense	H69 cells	-	USA, China	[20]
*C. parvum*	miRNA	Host defense	mICcl2 cells	C57BL/6 mice	France	[21]
*C. parvum*	PTHM	Host defense	FHs 74 INT, HCT-8, IEC4.1 cells	Mice in vivo infection	USA	[22]
*C. parvum*	PTHM	Cell migration	FHs 74 INT, HCT-8 cells	-	USA, China	[23,24]
*C. parvum*	miRNA	Apoptosis; cancer	HT-29 and HCT-8 cells	-	Malaysia	[25]
*C. parvum*	lncRNA; PTHM	Host defense	IEC4.1, muINTEPI cells	C57BL/6J mice	USA	[26]
*C. parvum*	lncRNA	Cell adhesion	HCT-8 cells	-	China	[27]
*C. parvum*	PTHM	Host defense	FHs 74 INT, HCT-8 cells	-	USA, China	[28]
*C. parvum*	miRNA	Host defense	HCT-8 cells	-	China	[29]
*C. parvum*	miRNA	Host defense; apoptosis	HCT-8 cells	-	China	[30]
*C. parvum*	lncRNA	Host defense	IEC4.1, RAW264.7 cells	C57BL/6J mice	USA	[31]
*C. parvum*	miRNA	Cell cycle regulation; cancer	Bioinformatics (GEO)/in silico	-	UK, Saudi Arabia, India	[32]
*C. parvum*	circRNA	Apoptosis	HCT-8 cells	-	China	[33]
*C. parvum*	lncRNA; PTHM	Host defense	HCT-8, IEC4.1 cells	C57BL/6J mice	USA	[34]
*C. parvum*	PTHM	Host defense; cancer	HCT-8 cells	CB17-SCID mice	France, Belgium	[35]
*C. parvum*	miRNA	Host defense; cell proliferation; apoptosis	HCT-8 cells	-	Turkey	[36]
*C. parvum*	miRNA	Apoptosis	HCT-8 cells	-	China	[37]
*P. berghei*	miRNA	Host defense	-	C57BL/6 and CBA mice	Australia, Denmark	[38]
*P. berghei*	miRNA	Host defense; cell proliferation; apoptosis	HEK293T cells	C57BL/6J and NMRI mice	Germany, USA	[39]
*P. berghei* and *P. yoelli*	miRNA	Host defense; cell adhesion; endocytosis; apoptosis	-	CBA mice	Spain, Australia, Peru	[40]
*P. chabaudi*	miRNA	Host defense; apoptosis	-	C57BL/6 mice	Germany, Saudi Arabia	[41]
*P. chabaudi*	miRNA	Host defense; cell cycle regulation; apoptosis; cancer	-	C57BL/6 mice	Saudi Arabia, Germany, Egypt	[42]
*P. chabaudi*	DNAm	Host defense	-	C57BL/6 mice	Saudi Arabia, Germany, Egypt	[43]
*P. chabaudi*	DNAm	Host defense; erythropoiesis	-	Balb/c mice	Saudi Arabia, Germany, Egypt, Spain	[44]
*P. chabaudi*	PTHM	Host defense	-	C57BL/6J mice	UK	[45]
*P. falciparum*	miRNA	Host defense	Human RBC, J2E, F4N, BW5147, J558L and 416B cells	-	UK	[46]
*P. falciparum*	miRNA	Host defense	Human RBC	-	USA, UK	[47]
*P. falciparum*	miRNA	Host defense	Human RBC	-	USA, Switzerland, UK	[48]
*P. falciparum*	miRNA	Host defense; cell communication	Human RBC	-	USA, Switzerland	[49]
*P. falciparum*	miRNA	Host defense	-	Artificial human infection	Australia	[50]
*P. falciparum*	DNAm	Drug resistance	-	Malaria patients	India	[51]
*P. falciparum*	miRNA	Host defense	Human RBC	-	China	[52]
*P. falciparum* and *P. vivax*	miRNA	Host defense; cell adhesion	-	Malaria patients	China	[53]
*P. falciparum*	PTHM	Host defense	-	Malaria patients	USA, Brazil, Netherlands	[54]
*P. falciparum*	miRNA	Host defense	Human RBC	-	India	[55]
*P. falciparum*	PTHM	Host defense		Artificial human infection	Netherlands, Denmark, Germany	[56]
*P. falciparum*	miRNA	Host defense	Human RBC and HEK 293T cells	-	India, Belgium	[57]
*P. falciparum* and *P. vivax*	miRNA	Host defense; cell adhesion	-	Malaria patients	Thailand, Australia	[58]
*P. falciparum*	miRNA	Host defense	-	Malaria patients	Germany	[59]
*P. falciparum*	miRNA	Host defense; cell proliferation; neural pathways	HBE cells	Malaria patients	Spain, Mozambique, USA	[60]
*P. falciparum*	miRNA	Host defense; cell adhesion; erythropoiesis	-	Malaria patients	Iran	[61]
*P. falciparum*	miRNA	Host defense	-	Malaria patients	USA, Ghana	[62]
*P. vivax*	miRNA	Host defense	-	Malaria patients	Thailand, Japan	[63]
*P. vivax*	miRNA	Host defense; erythropoiesis; apoptosis	-	Malaria patients	India, Qatar	[64]
*P. vivax*	miRNA	Host defense	-	Malaria patients	Brazil, Germany	[65]
*P. yoelli*	miRNA	Host defense; neural pathways	-	CBA mice	Australia, USA	[66]
*P. yoelli*	lncRNA	Host defense	-	BALB/c mice	China	[67]
*T. annulata*	PTHM	Cancer	BL3 cells	-	France	[68]
*T. annulata*	miRNA	Cancer	BL3 cells	-	France, USA	[69]
*T. annulata*	miRNA	Host defense; cancer	BL20 cells	-	UK	[70]
*T. annulata*	miRNA	Cancer	BL3,BL20 cells	-	Saudi Arabia, France, Japan	[71]
*T. gondii*	PTHM	Host defense	-	C57BL/6 mice	USA	[72]
*T. gondii*	PTHM	Host defense	-	C57BL/6 mice	USA	[73]
*T. gondii*	miRNA	Apoptosis	HFF, VERO cells	-	USA	[74]
*T. gondii*	PTHM	Host defense	RAW264.7, BMDM (BALB/c mice) cells	-	Germany	[75]
*T. gondii*	miRNA	Apoptosis	Human macrophage, THP-1 cells	-	China, Australia	[76]
*T. gondii*	miRNA	Host defense	-	Kunming mice	China, UK	[77]
*T. gondii*	miRNA	Apoptosis	Human macrophage, THP-1 cells	-	China	[78]
*T. gondii*	miRNA	Host defense	HFF cells	Swiss OF1, BALB/cJRj, C57BL/6JRj mice	France, USA	[79]
*T. gondii*	miRNA	Host defense; cell cycle regulation	-	BALB/c mice	China	[80]
*T. gondii*	miRNA	Neural pathways	-	BALB/c mice	USA, China	[81]
*T. gondii*	miRNA	Neural pathways	SK-N-MC cells	CD-1 mice	USA, China	[82]
*T. gondii*	DNAm	Reproductive pathways	-	C57Bl/6 mice	Czech Republic, Germany	[83]
*T. gondii*	DNAm	Neural pathways	-	Wistar rats	Singapore	[84]
*T. gondii*	PTHM	Host defense	HFF, 293FT, HEK293 cells	-	USA	[85]
*T. gondii*	miRNA	Host defense; apoptosis; cancer	-	Kunming mice	China	[86]
*T. gondii*	miRNA	Cell cycle regulation; cell proliferation	L6 cells	-	Korea	[87]
*T. gondii*	PTHM	Host defense	HFF, RAW264.7, 2fTGH, U3A, HEK-Blue IFN-γ cells	BALB/cJRj mice	France	[88]
*T. gondii*	PTHM	Host defense	RAW 264.7, U3A, U3A-STAT1, HeLa cells	CD-1, C57BL/6 mice	USA	[89]
*T. gondii*	miRNA	Host defense; apoptosis	-	Chinese Li Hua domestic cats (*Felis catus*)	China	[90]
*T. gondii*	miRNA	Neural pathways	L-NSC, S-NSC, human monocytic cells	Toxoplasmosis patients	USA, UK, Canada, Australia	[91]
*T. gondii*	DNAm	Neural pathways	WERI-Rb-1 cells	-	Australia	[92]
*T. gondii*	miRNA	Host defense; cancer	-	BALB/c mice	China, UK	[93]
*T. gondii*	lncRNA	Host defense	HFF, THP-1 cells	-	China	[94]
*T. gondii*	lncRNA	Host defense	BMDM (C57BL/6 mice)	-	USA	[95]
*T. gondii*	miRNA	Apoptosis	Human macrophage	-	Iran	[96]
*T. gondii*	PTHM	Host defense	BMDM (C57BL/6 mice), HFF, 293-T-Rex cells	BALB/cJRj mice	France	[97]
*T. gondii*	PTHM	Host defense	HFF cells	C57BL/6 mice	Belgium, Czech Republic	[98]
*T. gondii*	miRNA	Host defense; apoptosis	-	Pigs (*Sus scrofa*)	China	[99]
*T. gondii*	miRNA	Host defense; apoptosis	DC2.4, HFF cells	-	China	[100]
*T. gondii*	miRNA	Host defense; cell proliferation; apoptosis	HFF, porcine alveolar macrophages cells	-	China	[101]
*T. gondii*	miRNA	Host defense	Primary human retinal pigment epithelial cells, ARPE-19 cells	-	Australia, USA	[102]
*T. gondii*	lncRNA	Host defense; cell proliferation; cancer	Primary retinal Müller cells and MIO-M1 cells	-	Australia	[103]
*T. gondii*	miRNA	Host defense	Mouse macrophage (C57BL/6J mice)	-	China	[104]
*T. gondii*	miRNA	Host defense; apoptosis; cell cycle regulation	Human placental explants	-	Chile	[105]
*T. gondii*	miRNA	Host defense	-	Ocular toxoplasmosis patients	Brazil	[106]
*T. gondii*	miRNA	Host defense	-	Toxoplasmosis and AIDS patients	Brazil	[107]
*T. gondii*	miRNA	Neural pathways	-	C57BL/6J mice	Australia	[108]
*T. gondii*	circRNA; miRNA	Host defense; cell proliferation; erythropoiesis; cancer	-	BALB/c mice	China	[109]
*T. gondii*	PTHM; DNAm	Host defense	RAW264.7 cells	-	Germany	[110]
*T. gondii*	PTHM; DNAm	Cell cycle regulation	BeWo, U-118MG cells	-	France	[111]
*T. gondii*	PTHM	Host defense	-	C57BL/6J mice	USA, Italy	[112]
*T. gondii*	DNAm	Neural pathways	-	Wistar rats	Singapore	[113]
*T. gondii*	miRNA	Host defense	-	Swiss Albin mice	Egypt	[114]
*T. gondii*	miRNA	Host defense	-	A/Sn mice	Brazil	[115]
*T. gondii*	lncRNA	Apoptosis	BMDM (C57BL/6 mice)	-	USA	[116]
*T. gondii*	miRNA	Host defense	VERO cells	-	Brazil	[117]
*T. gondii*	PTHM	Host defense; cell proliferation	3D4/21, PK-15 cells	-	China	[118]
*T. gondii*	miRNA	Host defense; cell proliferation; apoptosis; cancer	-	Pigs (*Sus scrofa*)	China	[119]
*T. gondii*	miRNA	Host defense	-	Pigs (*Sus scrofa*)	China	[120]
*T. gondii*	miRNA	Host defense	DC2.4, HFF, RAW264.7 cells	-	China	[121]
*T. gondii*	miRNA	Cell proliferation	BV2, U87, U118 cells	C57BL/6 mice	Korea	[122]
*T. gondii*	lncRNA	Host defense	HFF cells	-	China, UK	[123]
*T. gondii*	lncRNA	Host defense	-	BALB/c, Kunming mice	China	[124]
*E. papillata*	miRNA	Cancer; host defense; apoptosis	-	Balb/c mice	Saudi Arabia, Germany, Egypt	[125,126]

circRNA: circular RNA; PTHM: post-translational histone modification; DNAm: DNA methylation. The cell line names are listed in Supplemental Table S1.

**Table 2:** Percentage of studies and the relative number of each parasite species or genus, regulatory mechanism, target process, and research model.

Parasites		Regulatory Mechanism		Targeted Process		Research Model	
Specie or Genus	Occurrence	Mechanism	Occurrence	Pathway	Occurrence	Model	Occurrence
*T. gondii*	53/116; 45.7%	ncRNA	88/116; 75.9%	Host defense	87/116; 75%	in vitro	72/116; 62.1%
*Plasmodium* spp.	30/116; 25.8%	miRNA	76/88; 86.4%	Apoptosis	23/116; 19.8%	in vivo	63/116; 54.3%
*C. parvum*	27/116;23.3%	lncRNA	11/88; 12.5%	Cancer	14/116; 12%		
*T. annulata*	4/116; 3.5%	circRNA	2/88; 2.3%	Cell proliferation	11/116; 9.5%		
*E. papillata*	2/116; 1.8%	PTHM	23/116; 19.8%	Neural pathways	9/116;7.8%		
		DNAm	9/116; 7.8%	Cell cycle regulation	6/116; 5.2%		
				Cell adhesion	5/116; 4.3%		
				Erythropoiesis	4/116; 3.4%		
				Cell migration	2/116; 1.7%		
				Cell comunication	1/116; 0.9%		
				Reproductive pathway	1/116; 0.9%		

circRNA: circular RNA; PTHM: post-translational histone modification; DNAm: DNA methylation.

**Table 3:** Epidrugs that were FDA-approved or tested in clinical trials in the selected studies.

Parasite	Pathway	Compound	Compound Analogous in Clinical Trial					Ref.
			Compound	Phase	Disease	Number	Status	
*T. gondii*	miRNA	anti-miR21	RG012	1	AS	NCT03373786	Completed	[122]
*T. gondii*	PTHM	MS-275	Entinostat (MS-275)	3	BC	NCT02115282	Active	[75]
*T. gondii*	DNAm	L-methionine	SAMe	4	RMD	NCT04832178	Active	[84]
*T. gondii*	PTHM	HDAC2- SiRNA	Vorinostat; romidepsin	FDA approved	CTCL	-	-	[118]
*T. annulata*	miRNA	anti-miR- 155	Cobomarsen (MRG-106)	2	CTCL	NCT02580552	Complete	[69]

PTHM: post-translational histone modification; DNAm: DNA methylation; AP: Alport syndrome; BC: breast cancer; CTCL: cutaneous T cell lymphoma; RMD: resistant major depression; SAMe: S-adenosylmethionine.

## Data Availability

No new data were created or analyzed in this study. Data sharing is not applicable to this article.

## Supplementary Materials

The following supporting information can be downloaded at: https://www.mdpi.com/article/10.3390/pathogens12020299/s1, Table S1: Abbreviation list; Table S2: Studies that modulate the epigenetic pathways interfered by parasites [148].
