# Supplementary material for: A Systematic Review of Apicomplexa Looking into Epigenetic Pathways and the Opportunity for Novel Therapies
Source: Pathogens. 2023 Feb 11;12(2):299. doi: 10.3390/pathogens12020299 (PMC9963874; doi:10.3390/pathogens12020299)
Supplement: Supplementary file 1 [file pathogens-12-00299-s001.zip › pathogens-2154035-supplementary.pdf]

Supplemental Table S1. Abbreviation list

Abbreviation	Definition
DNMT	DNA methyltransferase
DNMTi	DNA methyltransferase inhibitor
PTHM	Post-translational histone modification
ncRNA	non-coding RNA
siRNA	small-interfering RNA
miRNA	micro-RNA
lncRNA	long-non coding RNA
piRNA	piwi-RNA
circRNA	circular RNA
HDAC	Histone desacetylase
HDACi	Histone desacetylase inhibitor
HAT	Histone acetyltransferase
HMT	Histone methyltransferase
HMTi	Histone methyltransferase inhibitor
HDM	Histone desmethylase
HDMi	Histone desmethylase inhibitor
TgIST	T. gondii inhibitor of STAT1 transcriptional activity
STAT-1	Signal Transducer and Activator of Transcription 1
Mi-2/NuRD	Mi-2/nucleosome remodeling and deacetylase
TEEGR	Toxoplasma E2F4-associated EZH2-inducing gene regulator
EZH2	Enhancer of zeste 2 polycomb repressive complex 2 Subunit
PI3K/AKT/mTOR	phosphoinositide 3 kinase /Akt/mammalian (or mechanistic) target of rapamycin
UHRF1	ubiquitin-like containing PHD RING finger domain
ROP16	Ropthry 16
CCNB1	Cyclin B1 gene
TLR	Tool-like receptor
JAK/STAT	Janus kinase/signal transducers and activators of transcription
CX3CL1	C-X3-C Motif Chemokine Ligand 1
GP130	Glycoprotein 130/interleukin 6 signal transducer
ABCB1	ATP-binding cassette transporters
CREB1	cAMP responsive element binding protein 1
CREM	cAMP Responsive Element Modulator Signaling 1
SOCS1	suppressor of cytokine Signaling 1
DET-1	light-mediated developmental protein
CAV-1	Caveolin-1
ATF2	Activating transcription factor 2
PfEMP1	P. falciparum erythrocyte membrane protein-1
PKA-R	cAMP-dependent protein kinase
CIS	Cytokine-inducible Src homology 2-containing
SIRT1	Sirtuin-1
IκBα	NF-kappa-B inhibitor alpha
Cells lineages	

293FT	Human embryonal kidney cells
293-T-Rex	293 cells that express the tetracycline repressor protein
2fTGH	Human fiborsarcoma
3D4/21	Porcine alveolar macrophages cells
603B	Normal mouse biliary epithelial cells
ARPE19	Human retinal pigment epithelium
BeWo	Human trophoblast cell line
BL20	Bovine lymphosarcoma cell line
BL3	Bovine B lymphosarcoma cell line
BMDM	Bone marrow-derived macrophages
BMEC-1	Human bone marrow microvascular endothelial cell line
BV2	Murine microglial cells
Caco-2	Human colon adenocarcinoma
DC2.4	Mouse dendritic cell line
FHs 74 INT	Human colon cell line
GEO	Gene expression omnibus
H69	Human colangiocyte cell linage
HBE	Human brain endothelial cells
HCT-8	Human ileocecal adenocarcinoma
HEK293T	Human kidney cancer cell line
HEK-Blue IFN-γ cells	Human HEK293 cells - IFN-γ reporter cells
HeLa	Human cervical adenocarcinoma cells
HFF	Human foreskin fibroblast
HIBEpiC	Human intrahepatic biliary epithelial cells
HT-29	Colon adenocarcinoma cell line
IEC4.1	Murine intestinal epithelial cell line
Jukart	T lymphoblast
L6	Rat skeletal muscle cells
mICcl2	Intestinal crypt epithelial cell
MIO-M1	Human Muller cells
muINTEPI	Murine intestinal epithelial cell line
PK-15	Porcine kidney epithelial cells
RAW264.7	Murine leukemia macrophage cell line
RBC	Red blood cells
SK-N-MC	Human neuroepithelioma cell
SW480	Human colon adenocarcinoma
TBL20	Bovine lymphosarcoma cell line infected by T. annulata
TBL3	Bovine lymphosarcoma cell line infected by *T. annulata*
THP-1	Human monocyte
U-118MG	Human astrocytic cell line
U3A	Human fibrosarcoma
U87, U118	Human glioma cell lines
VERO cells	African green monkey kidney epithelial cells
WERI-Rb-1	Retinoblastoma cell line

Supplemental Table S2. Studies that modulate the epigenetic pathways interfered by parasites.

Parasite	Model	Parasite action	Chemical	Analogous chemical in clinical trial	Mechanism	Target	Outcome	Ref.
C. parvum	H69 cells	Decrease let7i	Anti-let7i	No	Increase let7i	TLR4 mRNA	Increase TLR/NFkB pathway; Decrease parasite burden	[11]
C. parvum	H69 cells	Decrease let7i	let7i precursor	No	Increase let7i	SNAP23 mRNA	Increase exosome release	[19]
C. parvum	HCT8 cells	Decrease let7i	let-7i precursor	No	Increase let7-i	SIRT1 mRNA	Decrease SIRT1	[20]
C. parvum	H69 cells	Decrease mir-98	miR-98 precursor	No	Increase miR-98	CIS mRNA	Increase NFkB pathway	[15]
C. parvum	H69 cells	Decrease mir-98	miR-98 precursor	No	Increase miR-98	SOCS4 mRNA	Increase STAT-3 and STAT-6 fosforilation	[16]
C. parvum	H69 and Jurkat cells	Decrease mir-513	miR-513 precursor	No	Increase miR-513	B7-H1 mRNA	Decrease Jurkat apoptosis	[13]
C. parvum	H69 and Jukart cells	Decrease mir-221	miR-221 precursor	No	Increase miR-221	ICAM-1 mRNA	Decrease adherence of Jurkat cells	[17]
C. parvum	HCT8 cells	Increase mir-942; decrease IFI27	miR-942-5p inhibitor	No	Decrease miR-942-5p	IFI27 mRNA	Increase IFI27; Increase apoptosis; Decrease parasite burden	[37]
C. parvum	HCT8 cells	Increase ciRS-7;	ciRS-7 vector	No	Increase ciRS-7	miR-1270	Decrease miR-1270; Increase RELA and NFκB pathway	[37]
C. parvum	HCT8 cells	Decrease mir-1270;	miR-1270 SiRNA	No	Decrease miR-1270	RELA mRNA	Increase RELA and NFκB pathway	[33]
C. parvum	IEC4.1 cells	Increase lncRNA Nostrill	Nostrill	No	Increase Nostrill; increase association NFκB to *ifr7* promoter	*IRF7* gene	Increase IRF7	[34]
C. parvum	IEC 4.1 and enteroids	Increase lncRNA NR_045064	NR_045064 SiRNA	No	Decrease NR_045064; Decrease WRD5/P300/H3K4 methyltransferase to *NOS2* and *CSF2* promoter	*NOS2* and *CSF2* genes	Decrease H3K4me1; Decrease NOS2 and CSF2	[26]
C. parvum	IEC4.1 cells	Increase lncRNA XR_001779380	SiRNA XR_001779380	No	Decrease XR_001779380	Stat1/Swi/Snf complex	Decrease NO2	[31]
C. parvum	HCT8 cells	Increase H3K9me3 mark	SiRNA PMDR1	No	Decrease PRDM1/G9a in *IL33, SLC7A8, LRP5* promoter	*IL33, SLC7A8, LRP5* genes	Decrease IL33, SLC7A8, LRP5	[32]
P. falciparum	RBC	Translocation of miRNA from host to parasite	miR-150-3p/ miR-197-5p mimic	No	Increase miR150-3p/miR-197-5p	PfApicortin mRNA	Decrease PfApicortin and parasite burden	[57]
P. falciparum	RBC	Translocation of miRNA from host to parasite	miR-451 mimic	No	Increase miR-451	PKA-R mRNA	Decrease reproduction and parasite burden	[47]
P. falciparum	RBC	Increase EV secretion containing mR-451/ miR-140	miR-451/-140 mimic	No	Increase miR-451/miR-140	PfEMP1 mRNA	Decrease *var* genes expression	[52]

P. falciparum	C57BL/6J mice injection	Increase mir-155	AAV8 miR-155	No	Increase miR-155	IFNγ pathway	Increase host protection	[39]
T. gondii	DC2.4; RAW264.7 cells	Increase miR-155 in DC2.4 cell exosomes	miR-155 mimic	No	Increase miR-155	SOCS1 mRNA	Macrophage M1 polarization; Decrease parasite proliferation	[121]
T. gondii	Human macrophage	Increase miR20a	Anti - miR20a	No	Decrease miR-20a	Apoptosis	Increase apoptosis	[96]
T. gondii	C57BL/6J mice macrophage	Decrease miR-187	miR-187 mimic	No	Increase miR-187	NFKBIZ mRNA	Increase IL-12	[104]
T. gondii	BV2, U87 cells	Increase miR-21 in BV2 cell exosomes	Anti-miR21	RG012	Decrease miR-21	FoxO1 mRNA	Decrease FoxO1 and p27; Decrease U 87cells proliferation	[148]
T. gondii	HFF; THP-1 cells	Increase lncRNA NONHSAT022487	ShRNA NONHSAT022487	No	Decrease NONHSAT022487	UNC93B1	Increase UNC93B1; Increase IL-12, IL-1β, IFNγ,TNFα	[94]
T. gondii	RAW264.7 cells	Decrease histone modifications and IFN-γ-responsive genes expression	TSA	Vorinostat; Valproic acid; Romidepsin; Entinostat (MS-275); Sodium butyrate	Decrease histone deacetylation	IFNγ induced genes	No change in HDAC activity and IFNγ genes expression	[110]
T. gondii	BMDM cells	Decrease histone acetylation and IFN-γ-responsive genes expression	Sodium butyrate and MS-275	Vorinostat; Valproic acid; Romidepsin; Entinostat (MS-275); Sodium butyrate	Decrease histone deacetylation	HDAC	Increase H2A/E and CIITA	[75]
T. gondii	HFF; HEK293 cells	Decrease IFN-γ responsive genes expression	TSA, MC1568, MS-275, and sodium butyrate	Vorinostat; Valproic acid; Romidepsin; Entinostat (MS-275); Sodium butyrate	Decrease histone deacetylation	IFNγ induced genes	No change in IFNγ genes expression	[85]
T. gondii	3D4/21; PK-15 cells	Increase HDAC2 and histone Decrotonylation	HDAC2 SiRNA	Vorinostat; Valproic acid; Romidepsin; Entinostat (MS-275); Sodium butyrate	Decrease histone deacrotonylation	HDAC2	Increase crotonylation, NFκB, and cell proliferation via PI3K/AKT	[118]
T. gondii	Wistar rats	Hypomethylation of *Avp* gene in MePD	L-methionine	L-methionine; SAMe	Global hypermethylation	*AVP* gene	Restore normal behaviour	[84]
T. annulata	TBL3 cells	Increase miR-155	Anti-miR- 155	Cobomarsen (MRG-106)	Decrease miR-155	DET1	Increase DET1; decrease cell proliferation	[69]
T. annulata	TBL20 cells	Increase miR126-5p	Anti-miR126-5p	No	Decrease miR126-5p	JIP2	Decrease MMP9	[71]

REFERENCES

11. Chen, X.-M.; Splinter, P.L.; O'Hara, S.P.; LaRusso, N.F. A Cellular Micro-RNA, Let-7i, Regulates Toll-like Receptor 4 Expression and Contributes to Cholangiocyte Immune Responses against Cryptosporidium Parvum Infection. *J. Biol. Chem.* **2007**, *282*, 28929–28938, doi:10.1074/jbc.M702633200.

13. Gong, A.-Y.; Zhou, R.; Hu, G.; Liu, J.; Sosnowska, D.; Drescher, K.M.; Dong, H.; Chen, X.-M. Cryptosporidium Parvum Induces B7-H1 Expression in Cholangiocytes by Down-Regulating MicroRNA-513. *J. Infect. Dis.* **2010**, *201*, 160–169, doi:10.1086/648589.

15. Hu, G.; Zhou, R.; Liu, J.; Gong, A.-Y.; Eischeid, A.N.; Dittman, J.W.; Chen, X.-M. MicroRNA-98 and Let-7 Confer Cholangiocyte Expression of Cytokine-Inducible Src Homology 2-Containing Protein in Response to Microbial Challenge. *J. Immunol.* **2009**, *183*, 1617–1624, doi:10.4049/jimmunol.0804362.

16. Hu, G.; Zhou, R.; Liu, J.; Gong, A.-Y.; Chen, X.-M. MicroRNA-98 and Let-7 Regulate Expression of Suppressor of Cytokine Signaling 4 in Biliary Epithelial Cells in Response to Cryptosporidium Parvum Infection. *J. Infect. Dis.* **2010**, *202*, 125–135, doi:10.1086/653212.

17. Gong, A.-Y.; Hu, G.; Zhou, R.; Liu, J.; Feng, Y.; Soukup, G.A.; Chen, X.-M. MicroRNA-221 Controls Expression of Intercellular Adhesion Molecule-1 in Epithelial Cells in Response to Cryptosporidium Parvum Infection. *Int. J. Parasitol.* **2011**, *41*, 397–403, doi:10.1016/j.ijpara.2010.11.011.

19. Hu, G.; Gong, A.-Y.; Roth, A.L.; Huang, B.Q.; Ward, H.D.; Zhu, G.; LaRusso, N.F.; Hanson, N.D.; Chen, X.-M. Release of Luminal Exosomes Contributes to TLR4-Mediated Epithelial Antimicrobial Defense. *PLoS Pathog.* **2013**, *9*, e1003261, doi:10.1371/journal.ppat.1003261.

20. Xie, H.; Lei, N.; Gong, A.-Y.; Chen, X.-M.; Hu, G. Cryptosporidium Parvum Induces SIRT1 Expression in Host Epithelial Cells through Downregulating Let-7i. *Hum. Immunol.* **2014**, *75*, 760–765, doi:10.1016/j.humimm.2014.05.007.

22. Wang, Y.; Gong, A.-Y.; Ma, S.; Chen, X.-M.X.; Strauss-Soukup, J.K.; Chen, X.-M.X. Delivery of Parasite Cdg7_Flc_0990 RNA Transcript into Intestinal Epithelial Cells during Cryptosporidium Parvum Infection Suppresses Host Cell Gene Transcription through Epigenetic Mechanisms. *Cell. Microbiol.* **2017**, *19*, e12760, doi:10.1111/cmi.12760.

26. Li, M.; Gong, A.-Y.; Zhang, X.-T.; Wang, Y.; Mathy, N.W.; Martins, G.A.; Strauss-Soukup, J.K.; Chen, X.-M. Induction of a Long Noncoding RNA Transcript, NR_045064, Promotes Defense Gene Transcription and Facilitates Intestinal Epithelial Cell Responses against Cryptosporidium Infection. *J. Immunol.* **2018**, *201*, 3630–3640, doi:10.4049/jimmunol.1800566.

31. Gong, A.-Y.; Wang, Y.; Li, M.; Zhang, X.-T.; Deng, S.; Chen, J.M.; Lu, E.; Mathy, N.W.; Martins, G.A.; Strauss-Soukup, J.K.; et al. LncRNA XR_001779380 Primes Epithelial Cells for IFN-γ-Mediated Gene Transcription and Facilitates Age-Dependent Intestinal Antimicrobial Defense. *MBio* **2021**, *12*, e0212721, doi:10.1128/mBio.02127-21.

33. Yin, Y.-L.; Liu, T.-L.; Yao, Q.; Wang, Y.-X.; Wu, X.-M.; Wang, X.-T.; Yang, X.; Song, J.-K.; Zhao, G.-H. Circular RNA CiRS-7 Affects the Propagation of Cryptosporidium Parvum in HCT-8 Cells by Sponging MiR-1270 to Activate the NF-KB Signaling Pathway. *Parasit. Vectors* **2021**, *14*, 238, doi:10.1186/s13071-021-04739-w.

34. Mathy, N.W.; Deng, S.; Gong, A.-Y.; Li, M.; Wang, Y.; Burleigh, O.; Kochvar, A.; Whiteford, E.R.; Shibata, A.; Chen, X.-M. The Long Non-Coding RNA Nostrill Regulates Transcription of Irf7 Through Interaction With NF-KB P65 to Enhance Intestinal Epithelial Defense Against Cryptosporidium Parvum. *Front. Immunol.* **2022**, *13*, 863957, doi:10.3389/fimmu.2022.863957.

37. Xie, F.; Zhang, Y.; Li, J.; Sun, L.; Zhang, L.; Qi, M.; Zhang, S.; Jian, F.; Li, X.; Li, J.; et al. MiR-942-5p Targeting the IFI27 Gene Regulates HCT-8 Cell Apoptosis via a TRAIL-Dependent Pathway during the Early Phase of Cryptosporidium parvum Infection. *Parasites Vectors* **2022**, *15*, 291. https://doi.org/10.1186/s13071-022-05415-3.

39. Hentzschel, F.; Hammerschmidt-Kamper, C.; Börner, K.; Heiss, K.; Knapp, B.; Sattler, J.M.; Kaderali, L.; Castoldi, M.; Bindman, J.G.; Malato, Y.; et al. AAV8-Mediated In Vivo Overexpression of MiR-155 Enhances the Protective Capacity of Genetically Attenuated Malarial Parasites. *Mol. Ther.* **2014**, *22*, 2130–2141, doi:https://doi.org/10.1038/mt.2014.172.

47. LaMonte, G.; Philip, N.; Reardon, J.; Lacsina, J.R.R.; Majoros, W.; Chapman, L.; Thornburg, C.D.D.; Telen, M.J.J.; Ohler, U.; Nicchitta, C.V. V; et al. Translocation of Sickle Cell Erythrocyte MicroRNAs into Plasmodium Falciparum Inhibits Parasite Translation and Contributes to Malaria Resistance. *Cell Host Microbe* **2012**, *12*, 187–199, doi:https://doi.org/10.1016/j.chom.2012.06.007.

52. Wang, Z.; Xi, J.; Hao, X.; Deng, W.; Liu, J.; Wei, C.; Gao, Y.; Zhang, L.; Wang, H. Red Blood Cells Release Microparticles Containing Human Argonaute 2 and MiRNAs to Target Genes of Plasmodium Falciparum. Emerg. Microbes Infect. 2017, 6, 1–11, doi:10.1038/emi.2017.63.

57. Chakrabarti, M.; Garg, S.; Rajagopal, A.; Pati, S.; Singh, S. Targeted Repression of Plasmodium Apicortin by Host MicroRNA Impairs Malaria Parasite Growth and Invasion. Dis. Model. Mech. 2020, 13, doi:10.1242/dmm.042820.

69. Marsolier, J.; Pineau, S.; Medjkane, S.; Perichon, M.; Yin, Q.Y.; Flemington, E.; Weitzman, M.D.; Weitzman, J.B. OncomiR Addiction Is Generated by a MiR-155 Feedback Loop in Theileria-Transformed Leukocytes. *PLoS Pathog.* **2013**, *9*, e1003222, doi:10.1371/journal.ppat.1003222.

71. Haidar, M.; Rchiad, Z.; Ansari, H.R.; Ben-Rached, F.; Tajeri, S.; Latre De Late, P.; Langsley, G.; Pain, A. MiR-126-5p by Direct Targeting of JNK-Interacting Protein-2 (JIP-2) Plays a Key Role in Theileria-Infected Macrophage Virulence. *PLoS Pathog.* **2018**, *14*, e1006942, doi:10.1371/journal.ppat.1006942.

75. Lang, C.; Hildebrandt, A.; Brand, F.; Opitz, L.; Dihazi, H.; Luder, C.G.K. Impaired Chromatin Remodelling at STAT1-Regulated Promoters Leads to Global Unresponsiveness of Toxoplasma Gondii-Infected Macrophages to IFN-Gamma. *PLOS Pathog.* **2012**, *8*, doi:10.1371/journal.ppat.1002483.

84. Hari Dass, S.A.; Vyas, A.; Dass, S.A.H.; Vyas, A. Toxoplasma Gondii Infection Reduces Predator Aversion in Rats through Epigenetic Modulation in the Host Medial Amygdala. *Mol. Ecol.* **2014**, *23*, 6114–6122, doi:10.1111/mec.12888.
85. Rosowski, E.E.; Nguyen, Q.P.; Camejo, A.; Spooner, E.; Saeij, J.P.J. Toxoplasma Gondii Inhibits Gamma Interferon (IFN-Gamma)- and IFN-Beta-Induced Host Cell STAT1 Transcriptional Activity by Increasing the Association of STAT1 with DNA. *Infect. Immun.* **2014**, *82*, 706–719, doi:10.1128/IAI.01291-13 WE - Science Citation Index Expanded (SCI-EXPANDED).
94. Liu, W.; Huang, L.; Wei, Q.; Zhang, Y.; Zhang, S.; Zhang, W.; Cai, L.; Liang, S. Microarray Analysis of Long Non-Coding RNA Expression Profiles Uncovers a Toxoplasma-Induced Negative Regulation of Host Immune Signaling. *Parasit. Vectors* **2018**, *11*, 174, doi:10.1186/s13071-018-2697-8.
96. Rezaei, F.; Daryani, A.; Sharifi, M.; Sarvi, S.; Jafari, N.; Pagheh, A.S.; Hashemi, N.; Hejazi, S.H. MiR-20a Inhibition Using Locked Nucleic Acid (LNA) Technology and Its Effects on Apoptosis of Human Macrophages Infected by Toxoplasma Gondii RH Strain. *Microb. Pathog.* **2018**, *121*, 269–276, doi:10.1016/j.micpath.2018.05.030.
104. Jiang, H.; Zhai, T.; Yu, Y.; Li, X.; Gong, P.; Zhang, X.; Li, G.; Li, J. Delayed IL-12 Production by Macrophages during Toxoplasma Gondii Infection Is Regulated by MiR-187. *Parasitol. Res.* **2020**, *119*, 1023–1033, doi:10.1007/s00436-019-06588-0.
110. Nast, R.; Choepak, T.; Lüder, C.G.K.; Lueder, C.G.K.; Lüder, C.G.K. Epigenetic Control of IFN-γ Host Responses During Infection With Toxoplasma Gondii. *Front. Immunol.* **2020**, *11*, 581241, doi:10.3389/fimmu.2020.581241.
118. Yang, J.; He, Z.; Chen, C.; Li, S.; Qian, J.; Zhao, J.; Fang, R. Toxoplasma Gondii Infection Inhibits Histone Crotonylation to Regulate Immune Response of Porcine Alveolar Macrophages. *Front. Immunol.* **2021**, *12*, 696061, doi:10.3389/fimmu.2021.696061.
121. Jiang, D.; Wu, S.; Xu, L.; Xie, G.; Li, D.; Peng, H. Anti-Infection Roles of MiR-155-5p Packaged in Exosomes Secreted by Dendritic Cells Infected with Toxoplasma Gondii. *Parasit. Vectors* **2022**, *15*, 3, doi:10.1186/s13071-021-05003-x.
148. Jung, T.W.; Park, J.; Sun, J.L.; Ahn, S.H.; Abd El-Aty, A.M.; Hacimuftuoglu, A.; Kim, H.-C.; Shim, J.-H.; Shin, S.; Jeong, J.H. Administration of Kynurenic Acid Reduces Hyperlipidemia-Induced Inflammation and Insulin Resistance in Skeletal Muscle and Adipocytes. *Mol. Cell. Endocrinol.* **2020**, *518*, 110928, doi:10.1016/j.mce.2020.110928.
